# Use of Phytone Peptone to Optimize Growth and Cell Density of *Lactobacillus reuteri*

**DOI:** 10.3390/foods4030318

**Published:** 2015-08-10

**Authors:** Olabiyi A. Atilola, Rabin Gyawali, Sulaiman O. Aljaloud, Salam A. Ibrahim

**Affiliations:** 1 Food Microbiology and Biotechnology Laboratory, Agricultural and Technical State University, 173 Carver Hall, Greensboro, NC 27411, USA; E-Mails: omarbiyi@yahoo.com (O.A.A.); rgyawali@aggies.ncat.edu (R.G.); 2 Department of Exercise Physiology, College of Sport Sciences and Physical Activity King Saud University, P.O. Box 2458, Riyadh, 11451, Saudi Arabia; E-Mail: saljaloud@ksu.edu.sa

**Keywords:** cell density, growth, *L. reuteri*, phytone peptone, protein

## Abstract

The objective of this study was to determine the use of phytone peptone to optimize the growth and cell density of *Lactobacillus reuteri.* Four strains of *L. reuteri* (DSM 20016, SD 2112, CF 2-7F, and MF 2-3,) were used in this study. An overnight culture of individual strains was inoculated into fresh basal media with various protein sources (peptone, tryptone, proteose peptone #3, phytone peptone, tryptic soy broth, yeast extract, and beef extract). Samples were then mixed well and incubated at 37 °C for 15 h. Bacterial growth was monitored by measuring turbidity (optical density 610 nm) at different time intervals during the incubation period. At the end of incubation, samples were plated on de-Man Rogosa Sharpe (MRS) agar to determine the bacterial population. Our results showed that phytone peptone promoted the growth of *L. reuteri* (*p* < 0.05) by 1.4 log CFU/mL on average compared to the control samples. Therefore, phytone peptone could be included in laboratory media to enhance growth and increase the cell density of *L. reuteri*.

## 1. Introduction

*Lactobacillus reuteri* is used as a probiotic to promote health and improve the body’s defenses against medical conditions and illnesses such as gastrointestinal tract problems, atopic disease, colic, and oral gingivitis [1,2,3,4]. Although *L. reuteri* has a variety of beneficial effects on human health, it has had limited application in commercial dairy foods. Xanthopoulos *et al*. [5] found that *L. reuteri* had a low acidification ability compared to other tested strains. High cell density cultivations have become increasingly important as several companies have developed an interest in lactic acid bacteria (LAB) not simply as a starter culture but also as a valuable probiotic additive to their food products. It has been shown that high cell density is necessary in order to obtain the health benefits conferred by probiotics [6,7].

According to Fuller [8], probiotics are live microbial supplements that beneficially affect the host by improving its intestinal microbial balance. In our laboratory, we have defined growth-promoting factors for probiotic strains as protein sources that promote the growth of bacterial cells that impact probiotic functionality [9,10]. The functionality of probiotics, including lactic acid bacteria, is determined by the presence of growth-promoting factors. Lactic acid bacteria strains predominantly use peptides to meet their demand for complex nitrogen [11]. Van Niel and Hahn-Hägerdal [12] reported that peptides are either required for the growth of lactic acid bacteria or for the stimulation of their growth. Peptides can be derived from different sources, such as papain-digested skim milk, yeast extract, tryptone (trypsine-treated casein), soy peptones, peptones of animal origin, corn steep liquor, liver extracts, or whey protein hydrolysates [13]. Since each source contains different peptides, each strain will respond differently to each protein source because of the specificity of the enzymes involved. A review of the literature revealed very few studies examining the impact of extensive protein sources on *L. reuteri* or other lactic acid bacteria. 

Lactobacilli are extremely fastidious organisms that are usually grown in complex media such as deMan-Rogosa-Sharpe (MRS) [14]. Most phosphate-buffered media do not support the growth of lactobacilli as these media do not fulfill the amino acid requirement or they have a very low buffering capacity [15]. Recent efforts have focused on looking for effective nutritional sources for the growth of lactobacilli that depend on the composition of the media in which they are cultivated [16,17]. In addition, new fermentation techniques need to be employed to develop strains with improved physiology and functionality. These enhancements would increase the range of commercially available probiotics and expand product applications. The growth of *L. reuteri* is very limited and certain nutrients are needed for optimum growth and high cell density. Thus, a protein-rich medium with a high buffering capacity would support higher cell growth and cell densities of *Lactobacillus* spp. The objective of this study was to investigate the use of phytone peptone in a laboratory medium to optimize the growth and cell density of *Lactobacillus reuteri*.

## 2. Experimental Section

### 2.1. Bacterial Strains and Inoculum Preparation

Four strains of *Lactobacillus reuteri* (*L. reuteri* DSM 20016, SD 2112, CF 2-7F, and MF 2-3) obtained from BioGia, Raleigh, North Carolina, were used in this study. Overnight cultures were inoculated into MRS broth and incubated anaerobically at 37 °C until they reached the stationary phase. Bacterial cells were centrifuged at 2500× *g* for 10 min at 4 °C (Eppendorf centrifuge 5416 R) and washed twice in 0.9% w/v saline solution. The pellets were then resuspended in 0.9% w/v saline solution for 30 min. The bacterial culture was serially diluted and 100 µL of each appropriated dilution were added to different treatment groups, giving an initial inoculum level of 2.00 log CFU/mL. The samples were then incubated at 37 °C for 15 h in an anaerobic condition. 

### 2.2. Protein Source

Table 1 gives a description of protein sources that were used in this study. The chemicals listed were utilized as primary sources of protein during most of the microbiological studies. However, these protein sources can contain other nutrients at very low concentrations that are not considered to be a major factor in bacterial growth. Because yeast extract used in the growth of *L. reuteri* is expensive, the aim of this study was to identify the best protein source to replace yeast extract.

**Table 1:** Chemical description of protein sources used in this study.

Source	Description/Application	Suppliers
Peptone	Enzymatic digest of animal tissue, nitrogen in a form readily available to bacteria	Fisher Scientific
Tryptone	Pancreatic digest of casein is used as a nitrogen source for bacteria	Fisher Scientific
Proteose peptone #3	Enzymatic digest of protein	BD Difco
Phytone peptone	Papaic digest of soybean meal, designed specifically for cell culture applications, non-animal origin	BD Difco
Tryptic soy broth	Dehydrated media for isolation, detection, and cultivation of a wide variety of microorganisms	BD Difco
Yeast extract	Water soluble portion of autolyzed yeast containing vitamin B complex, used in preparing microbiological culture media	Fisher Scientific
Beef extract	Desiccated beef powder used for culture media	BD Difco

### 2.3. Preparation of Basal Medium (BM) for Protein Supplement

Each liter of the basal medium containing 10 g dextrose, 2.5 g yeast extract, 5 g Na-acetate, 2 g (NH_4_)_2_ citrate, 2 g K_2_HPO_4_, 0.20 g MgSO_4_·7H_2_O, 0.05 g MnSO_4_·4H_2_O, 0.1 g l-cysteine hydrochloride monohydrate, and 1 mL tween 80 and was prepared. To determine the influence of different protein sources on the growth and cell density of *L. reuteri*, 1.0% (w/v) of each protein source (peptone, tryptone, proteose peptone #3, phytone peptone, tryptic soy broth, yeast extract, and beef extract) was added individually to a basal medium. The initial pH values of all samples were approximately 6.5 ± 0.2.

### 2.4. Bacterial Enumeration

The growth of bacterial strains was monitored by measuring the optical density at different time intervals using a spectrophotometer (Spectronic 21 Milton Roy Spectrophotometer, Thermo Electron Scientific Co., Madison, WI, USA) at 610 nm. The uncultured media were used as blanks, and the initial and final populations were determined. At the end of the incubation period (15 h), a 1 mL sample was withdrawn from each inoculated sample and serially diluted in 0.9% saline water. Appropriate dilutions were then surface plated (100 µL) onto duplicate MRS agar. Colonies were counted as CFU/mL after incubation at 37 °C for 48 h.

During the exponential phase, nutrients are abundant and bacteria grow at their maximum rate known as maximum specific growth rate (μ_max_). Maximum specific growth rates per hour for the tested strains were determined during the exponential growth phase by measuring optical density values [18].

### 2.5. Determination of pH Values

The pH values of samples were measured using a model HI 8416 pH-meter (Hanna Instruments, Limena, Italy) at the beginning (0 h) and end (15 h) of the fermentation period.

### 2.6. Determination of Buffering Capacity

After stabilizing, the initial pH values of all protein-supplemented media (proteose peptone, peptone, tryptone, proteose peptone #3, phytone peptone, tryptic soy broth without dextrose, yeast extract, and beef extract) were determined. A standard 0.1 N of hydrochloric acid (HCl) was prepared for titration of all samples. Two aliquots (25 mL) of each sample were placed in 50-mL beakers, and 1 mL of HCl acid was titrated slowly into each sample. The pH reading was recorded until the pH of suspension reached 1.5–2 (the normal pH of the stomach). The buffering capacity was then determined mathematically using the formula given by Van Slyke [19].

$$\frac{d\text{B}}{d\text{pH}}=\;\frac{\left(\text{mL acid added}\right)\left(\text{normality of acid}\right)}{\left(\text{volume of sample}\right)\left(\text{pH change}\right)}$$

### 2.7. Statistical Analysis

All experiments were replicated at least three times in a randomized block design with different nutrients used as the blocking criterion. Replicates consisting of three batches of each strain were obtained. The data obtained was analyzed by Analysis of Variance (ANOVA) using SAS statistical software (SAS Institute Inc., Cary, NC, USA). Treatment means were compared at a 5% significance level.

## 3. Results and Discussion 

### 3.1. Growth and Cell Density of L. reuteri

**Figure 1:**
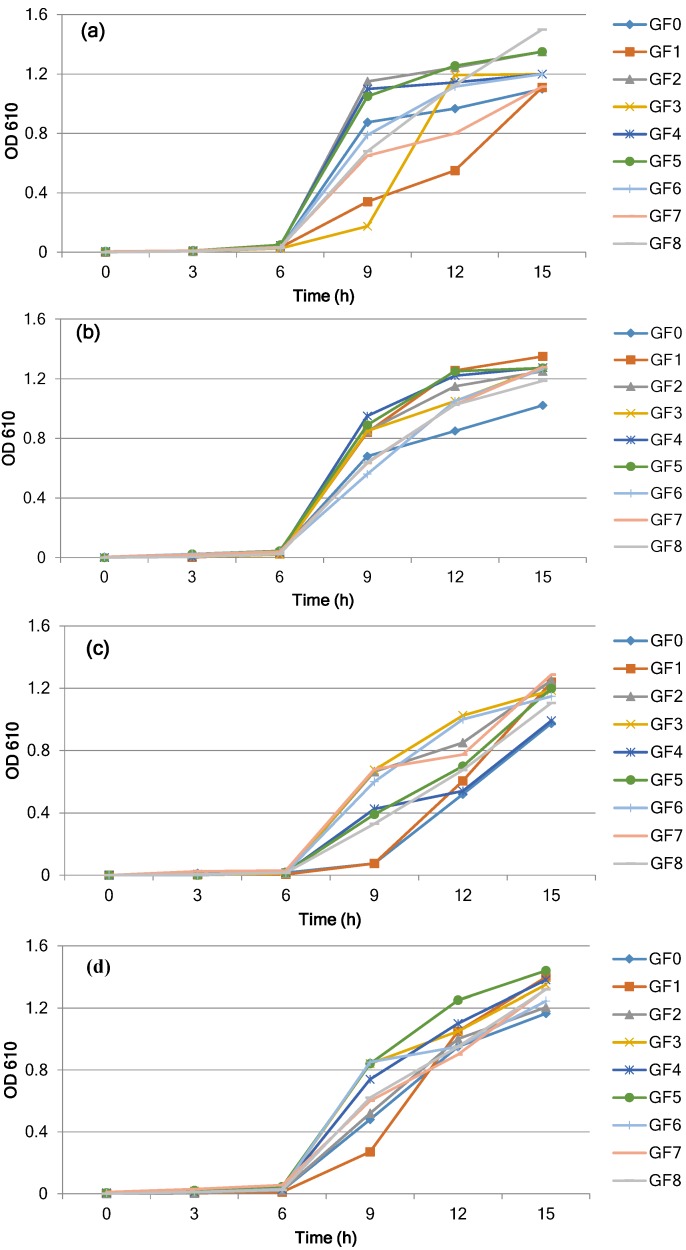
Changes in growth during incubation of *L. reuteri* strains (**a**) DSM 20016, (**b**) SD2112, (**c**) CF2-7F, and (**d**) MF2-3 at 37 °C in the basal medium with different protein sources: GF1-proteose peptone, GF2-peptone, GF3-tryptone, GF4-proteose peptone #3, GF5-phytone peptone, GF6-tryptic soy broth without dextrose, GF7-yeast extract, GF8-beef extract, GF0-control (no protein source).

Figure 1a–d shows the effect of different protein sources on the cell density and growth of four *L. reuteri* strains in a basal medium after 15 h of incubation at 37 °C. The bacterial strains continued to grow during the incubation period and reached the stationary phase within 15 h. In the control sample, the growth as observed by the turbidity and bacterial population reached 1.1 (O.D. 610 nm) and 8.40 log CFU/mL (Figure 1a, Table 2) for the *L. reuteri* strain DSM 20016. The turbidity readings and bacterial count for *L. reuteri* DSM 20016 reached a maximum absorbance of 1.35, and the population increased by 1.65 log CFU/mL and reached 10.05 log CFU/mL (*p* < 0.05) when GF5-phytone peptone was added (Figure 1a, Table 2). With the addition of other protein sources (GF3, GF6, and GF8), the population reached 9.52–9.92 log CFU/mL, indicating higher growth than that of the control. When basal media was supplemented with protein sources including GF1, GF2, GF4, and GF7, there was a slight but not significant (*p* > 0.05) growth compared to the control sample.

**Table 2:** Impact of different protein sources on pH values, growth (cfu/mL), and buffering capacity for *L. reuteri* strains during incubation at 37 °C for 15 h.

***L.reuteri* DSM 20016**									
Protein	GF0	GF1	GF2	GF3	GF4	GF5	GF6	GF7	GF8
pH	4.58 ± 0.17	4.69 ± 0.33	4.74 ± 0.22	4.78 ± 0.51	4.74 ± 0.32	4.69 ± 0.20	4.81 ± 0.12	4.74 ± 0.41	4.71 ± 0.33
Log CFU/mL	8.40 ± 0.31	8.59 ± 0.50	8.48 ± 0.14	9.59 ± 0.46^*^	8.48 ± 0.18	10.05 ± 0.12^*^	9.52 ± 0.21^*^	8.48 ± 0.38	9.68 ± 0.29^*^
μ_max_^a^	0.97 ± 0.19	0.55 ± 0.27	1.24 ± 0.33	1.19 ± 0.36	1.14 ± 0.19	1.26 ± 0.11	1.12 ± 0.30	0.80 ± 0.27	1.13 ± 0.14
Buffer capacity	8.39 ± 0.21	10.71 ± 0.33	10.31 ± 0.24	11.4 ± 0.31	10.31 ± 0.40	12.34 ± 0.10	11.86 ± 0.22	12.5 ± 0.23	10.31 ± 0.13
***L.reuteri* SD 2112**									
Protein	GF0	GF1	GF2	GF3	GF4	GF5	GF6	GF7	GF8
pH	4.73 ± 0.54	4.78 ± 0.11	4.88 ± 0.45	4.91 ± 0.11	4.83 ± 0.22	4.74 ± 0.14	4.89 ± 0.15	4.87 ± 0.51	4.81 ± 0.32
Log CFU/mL	8.57 ± 0.39	9.52 ± 0.26^*^	8.78 ± 0.12	9.72 ± 0.18^*^	9.06 ± 0.17	9.86 ± 0.15^*^	9.57 ± 0.34^*^	8.53 ± 0.28	9.92 ± 0.25^*^
μ_max_^a^	0.85 ± 0.33	1.26 ± 0.33	1.15 ± 0.33	1.05 ± 0.13	1.22 ± 0.10	1.25 ± 0.13	1.05 ± 0.39	1.03 ± 0.17	1.03 ± 0.22
Buffer capacity	8.39 ± 0.49	10.71 ± 0.36	10.31 ± 0.42	11.4 ± 0.26	10.31 ± 0.27	12.34 ± 0.11	11.86 ± 0.18	12.5 ± 0.22	10.31 ± 0.49
***L. reuteri* CF 2-7F**									
Protein	GF0	GF1	GF2	GF3	GF4	GF5	GF6	GF7	GF8
pH	4.48 ± 0.32	4.54 ± 0.33	4.72 ± 0.45	4.66 ± 0.27	4.51 ± 0.17	4.51 ± 0.34	4.61 ± 0.17	4.59 ± 0.49	4.53 ± 0.27
Log CFU/mL	8.48 ± 0.15	9.79 ± 0.19*	8.58 ± 0.31	9.64 ± 0.39*	8.91 ± 0.23	9.91 ± 0.33*	9.93 ± 0.19*	8.59 ± 0.17	9.83 ± 0.19*
μ_max_^a^	0.52 ± 0.19	0.61 ± 0.33	0.85 ± 0.33	1.03 ± 0.32	0.54 ± 0.25	0.70 ± 0.12	1.00 ± 0.14	0.78 ± 0.35	0.68 ± 0.12
Buffer capacity	8.39 ± 0.28	10.71 ± 0.34	10.31 ± 0.36	11.40 ± 0.22	10.31 ± 0.17	12.34 ± 0.11	11.86 ± 0.39	12.5 ± 0.33	10.31 ± 0.19
***L. reuteri* MF2-7F**									
Protein	GF0	GF1	GF2	GF3	GF4	GF5	GF6	GF7	GF8
pH	5.05 ± 0.31	4.89 ± 0.48	4.78 ± 0.21	5.12 ± 0.36	5.30 ± 0.31	5.02 ± 0.11	5.05 ± 0.15	4.90 ± 0.22	5.13 ± 0.43
Log CFU/mL	8.40 ± 0.45	9.52 ± 0.52	8.51 ± 0.24	9.45 ± 0.10	9.68 ± 0.32^*^	9.58 ± 0.23	9.54 ± 0.13	8.45 ± 0.15	9.40 ± 0.11
μ_max_^a^	0.95 ± 0.36	1.05 ± 0.45	1.00 ± 0.19	1.05 ± 0.18	1.10 ± 0.17	1.25 ± 0.43	0.95 ± 0.12	0.90 ± 0.18	0.95 ± 0.39
Buffer capacity	8.39 ± 0.19	10.71 ± 0.38	10.31 ± 0.36	11.4 ± 0.24	10.31 ± 0.20	12.34 ± 0.31	11.86 ± 0.17	12.5 ± 0.29	10.31 ± 0.18

All the values are presented as mean ± S.D (*n* = 3), * *p* ≤ 0.05, bacterial population is significantly different compared to the control (GF0), ^a^ Maximum specific growth rate.

In the control sample (GF0) for *L. reuteri* SD2112, the turbidity reading reached 1.02 and the bacterial population was 8.57 log CFU/mL after 15 h of incubation (Figure 1b, Table 2). With the addition of proteins GF5 and GF8, the turbidity reading reached maximum absorbance and the bacterial population reached 9.86 and 9.92 log CFU/mL (*p* < 0.05), respectively. Significant growth was also observed with other protein sources (GF1, GF3, GF6) when compared to the control, but the population was still lower than that of GF5 and GF8. However, basal media supplemented with GF2, GF4, and GF7 did not enhance the growth (*p* > 0.05) when compared to the control sample. 

Similarly, for *L. reuteri* CF2-7F (Figure 1c, Table 2), the turbidity readings and bacterial population reached 0.97 and 8.48 log CFU/mL in the control sample (GF0). The addition of protein sources to the basal media caused significant growth within 15 h (*p* < 0.05). The population reached a maximum of 9.83, 9.91, and 9.93 log CFU/mL with proteins GF8, GF5, and GF6, respectively. In the presence of proteins GF1 and GF3, the bacterial population continued to grow and reached 9.79 and 9.64 log CFU/mL, respectively. When compared to the control sample (GF0), there were no significant changes (*p* > 0.05) in bacterial population with protein sources GF2, GF4, and GF7. A similar result was observed with the addition of different protein sources for the *Lactobacillus* strain MF2 (Figure 1d, Table 2). The higher growth of 9.68 and 9.54 log CFU/mL was achieved in the presence of proteins GF4 and GF5 when compared to the control population (8.40 log CFU/mL).

Overall, the GF5 protein source exhibited the maximal cell count among the tested proteins. Alazzeh *et al*. [20] also studied the effect of different protein sources (beef extract, tryptic soy, tryptone, and yeast extract) on the enzyme production of *L. reuteri* strains. Yeast extract was found to be the best protein source to produce higher enzymes from *L. reuteri* strain CF2-7F. The maximum specific growth rate at 15 h (μ_max_) varied between 0.52 and 1.26 h^−1^ (Table 2). *L. rueteri* DSM20016 showed the best growth (10.05 log CFU/mL) among the strains tested and displayed a μ_max_ of 1.26 h^−1^ after 15 h of incubation, after which the growth rate decreased. In the control samples (GF0), all tested strains performed relatively lower than those with protein sources. We found a lower bacterial population, lower specific growth rate, and low buffering capacity. For all the proteins evaluated, our results showed that the basal medium with phytone peptone was significant in changing cell density growth (*p* < 0.5) for all tested strains of *L. reuteri*. The composition of phytone peptone was papaic digest of soybean meal (Difco, Becton Dickinson, Sparks, MD, USA). Soybeans are a significant source of protein and have been utilized in the food industry [21,22,23]. Moreover, soybeans are a cheap proteinaceous source and could be used as an alternative to yeast extract [23].

### 3.2. Changes in pH Values

Table 2 shows pH values measured at the end of the incubation period (15 h) for samples with different protein sources. During the incubation period, the pH value of the growth medium declined rapidly from an initial value of 6.5 to a final value of 4.48–5.30. The pH values ranged between 4.48–5.05 for the control and 4.51–5.30 for protein-supplemented samples. For the strains *L. reuteri* DSM 20016, *L. reuteri* SD 2112, and *L. reuteri* CF2-7F, pH values (4.69–4.81) were slightly higher in samples with protein compared to the control sample (4.48–4.73). Similarly, *L. reuteri* strain MF 2-3 showed slightly higher pH values, especially with protein sources GF3, GF4, and GF8 (5.12–5.30) when compared to the control sample (5.05). These slight changes in pH could be due to the different acidic activity of *Lactobacillus* strains. The differences in pH values might also be caused by the presence of only protein sources in the media. However, the presence of protein sources in the growth media could also result in a buffering capacity, and thus serve to prevent changes in pH values. In order to determine this effect, further study on the buffering capacity of different protein sources was conducted.

### 3.3. Buffering Capacity of Selected Protein Sources on L. reuteri 

Understanding buffering capacity is important in determining whether a pH change is due to the different protein sources used. Table 2 also summarizes the buffering capacity for different protein sources. For the control sample, the buffering capacity was observed at 8.39 mmol HCL. While the highest buffering capacity was observed at 12.5 and 12.34 for yeast extract and phytone peptone, respectively, little shift in pH with higher cell growth (CFU/mL) could be due to the buffering capacity of these protein sources. Many microbial organisms including *L. reuteri* are restricted to narrow pH limits; therefore, a culture medium that provides pH buffering is desirable. Protein sources having a higher buffering capacity can be used to maintain pH. This could provide protective mechanisms for the bacterial cells by preventing acid injury and, hence, allowing higher cell production. The relationship between cell growth and different proteins tested can be partly related to a high buffering capacity and ion exchange in the medium of some of the proteins. The few reports describing the effects of buffering capacity on microbial development have mainly focused on concentrated liquid milk products, which are considered good media for microbial growth [16,24]. Our results showed that phytone peptone and yeast extract have higher buffering capacities. However, based on cell growth, phytone peptone can be recommended as a good ingredient to develop media for the mass production of *L. reuteri.*

## 4. Conclusions

In this study, we demonstrated that phytone peptone has good potential to be added as a protein source for the growth and high cell mass of *L. reuteri* and has a high buffering capacity that possibly maintained the pH value during the fermentation process. Even though protein sources such as yeast and beef extract showed higher buffering capacities and high cell numbers, these ingredients are expensive and comprise a significant portion of media costs [18]. It has been estimated that yeast extract contributes >30% of the total production cost due to its high price [23]. Hence, the use of phytone peptone could be a less expensive alternative source for the cultivation of lactic acid bacteria, including *L. reuteri*. Phytone peptone is made of papaic digests of soybean meal/flour, a non-animal source, and could thus be used without labeling in food products. Early studies have demonstrated that certain peptides could act as growth-promoting factors for bifidobacteria known as bifidogenic factors. The presence of disulfides in these peptides is essential for the growth of several bifidobacteria [9]. Therefore, further work should be directed toward understanding the nature of peptides in the phytone peptone that contribute to the enhancement of *L. reuteri* growth. Additional work should also focus on the impact of protein sources in bulk media on the viability and enzymatic activities of *L. reuteri* in dairy products.
